# Use of a Questionnaire Measuring Anxiety About Alzheimer’s Disease and Implications for Improving Care

**DOI:** 10.1093/geroni/igaf122.459

**Published:** 2025-12-31

**Authors:** Tessa Lundquist

**Affiliations:** VA Boston Health Care System, Boston, Massachusetts, United States

## Abstract

Studies show that older adults have anxiety about Alzheimer’s disease (AD). There has been limited research utilizing a quantitative measure of this construct, and it is not known how anxiety about AD may impact wellbeing and health behavior. This presentation will review the use of a quantitative measure of AD anxiety and implications for improving support of older adults who worry about their memory. The AD Anxiety scale was administered to a sample of 43 older adults. The AD Anxiety scale exhibited adequate internal consistency (Cronbach’s alpha = .71). Participants had a mean AD Anxiety score of 19.89 (SD = 4.16, range 7-35). Participants who reported having concerns about their memory had significantly higher AD anxiety (M = 21.25, SD = 3.55; t(33) = 2.71, p < .05) than those who reported no such concerns (M = 18.11, SD = 3.45). AD Anxiety was distinct from more general state trait anxiety. Findings suggest that AD anxiety is a distinct form of anxiety and impacts participants with varying demographics differently. Research is mixed on how dementia worry may impact health related behavior. It will be important to determine if AD anxiety may be motivating for proactive health behavior or if it may lead to avoidance. Female participants had higher AD anxiety than males. Similarly, participants with a family history of dementia had more AD anxiety than those with no family history. Older adults’ demographics and previous experiences with dementia should be considered when addressing their AD anxiety for optimal health outcomes.

